# Illness Perception and Clinical Treatment Experiences in Patients with M. Maroteaux-Lamy (Mucopolysaccharidosis Type VI) and a Turkish Migration Background in Germany

**DOI:** 10.1371/journal.pone.0066804

**Published:** 2013-06-24

**Authors:** Hansjörg Dilger, Linn Leissner, Lenka Bosanska, Christina Lampe, Ursula Plöckinger

**Affiliations:** 1 Institute of Social and Cultural Antropology, Freie Universität Berlin, Berlin, Germany; 2 Centre of Excellence for Rare Metabolic Diseases, Interdisciplinary Centre of Metabolism, Campus Virchow-Klinikum, Charité-Universitätsmedizin Berlin, Berlin, Germany; 3 Department of Pediatric and Adolecscent Medicine, Villa Metabolica, University Medical Center Mainz, Mainz, Germany; Instituto de Ciencia de Materiales de Madrid - Instituto de Biomedicina de Valencia, Spain

## Abstract

**Introduction:**

Mucopolysaccharidosis VI (MPS VI) is an inherited lysosomal storage disease caused by a mutation of the gene for arylsulfatase B (ASB). Of the thirty-one patients registered in Germany, almost fifty percent have a Turkish migration background. MPS VI is treated by enzyme replacement therapy (ERT), which is time-consuming and expensive.

**Methods:**

This interdisciplinary study explored the illness perceptions and clinical treatment experiences among ten MPS VI patients with a Turkish migration background in two centers for metabolic diseases (Berlin and Mainz, Germany). The clinical treatment situation was observed and semi-structured interviews were conducted with patients and health care personnel, in addition to participatory observation in four patients' everyday environments in Berlin. The data from the interviews, patient records, and personal field notes were encoded, cross-related, and analyzed.

**Results:**

Patients' acknowledgement of the disease and coping strategies are influenced predominantly by the perception of their individual health status and the handling of the disease within their family. Patients' willingness to cooperate with treatment strategies is further modified by their knowledge of the disease and the relationships with their health care providers. In this analysis, cultural factors turned out to be marginally relevant.

**Conclusion:**

As with other chronic and debilitating diseases, effective treatment strategies have to reach beyond delivering medication. Health care providers need to strengthen the support for patients with a migration background. In this regard, they should respect the patients' cultural and social background and their personal perception of the disease and the therapy. Yet structural and social aspects (clinical setting, family and educational background) may be more crucial here than “cultural barriers.”

## Introduction

Mucopolysaccharidosis VI (MPS VI) is an inherited lysosomal storage disease (LSD) caused by a mutation of the arylsulfatase B (ASB) gene that results in reduced activity of the enzyme ASB. The incidence of MPS VI varies among different populations and geographic regions, from 1 in 238,000 (Northern Portugal) to 1 in 1,298,000 (British Columbia, Canada) [1] [2] [3] [4] [5]. The prevalence is estimated to be 1100 patients worldwide [6] [7]. Between 1980 and 1995 thirty-one cases were registered in Germany. Of these, about fifty percent were patients with a Turkish migration background [8].

MPS VI is inherited in an autosomal recessive pattern. The high prevalence in the Turkish population is due to consanguinity, i.e. intermarriage between cousins, which increases the incidence of MPS VI in families with a history of the disease [9]. The reduction of enzyme activity is variable, as is the clinical course of the disease, currently classified as rapidly or slowly progressing on the basis of their clinical phenotype and urinary glycosaminoglycan concentration. While the clinical presentation of patients with MPS VI demonstrates a continuum of signs and symptoms, a somewhat stronger genotype/phenotype correlation may be evident especially in cardiovascular manifestations [10] [11] [12].

The MPS VI phenotype comprises a severe skeletal dysplasia resulting in short stature and various bone deformations. Infiltrative cardiomyopathy and respiratory dysfunction are both due to the storage of glycosaminoglycan (GAG). Corneal opacity and atrophy of the optic nerve may result in amaurosis, and conductive and sensorineuronal hearing loss may lead to deafness. Coarse facial features, the deformation of hands and feet, as well as short stature are the visible manifestations of the disease and thus contribute to the mental distress of patients. MPS VI is not associated with cognitive impairment and is clinically heterogeneous (Table 1). There are large variations with regard to the age of onset, organ systems affected, and the severity of the disease. In addition, the rate of disease progression varies widely.

**Table 1:** Signs and symptoms of Maroteaux-Lamy syndrome (MPS VI).

Organ	Signs and symptoms
Ear, nose, throat and respiratory system	Hearing loss
	Recurrent ear infections
	Obstructive sleep apnoea
	Pneumonia
Eye	Corneal clouding
	Retinopathy
	Ocular hypertension and glaucoma
	Decreased visual acuity and blindness
	Optic nerve abnormalities
Heart and vasculature	Electrocardiographic abnormalities
	Valve disease
	Cardiomyopathy
	Coronary artery disease
	Endocarditis
	Systemic vascular narrowing and arterial
	hypertension
Bone and joints	Skeletal dysplasia
	Growth impairment
	Joint stiffness
	Degenerative arthritis
Central and peripheral nervous system	Carpal tunnel syndrome
	Communicating hydrocephalus
	Spinal cord compression

Since 2006, enzyme replacement therapy (ERT, Naglazyme ®) has been available for the treatment of patients with MPS VI. ERT has to be given intravenously, is generally well tolerated, and has shown to improve some aspects of MPS VI and may attenuate disease progression [13] [14].

ERT infusions take up to five hours per week. This is felt as an inappropriate burden by some patients and they fail to adhere to the treatment schedule. However, therapeutic efficacy may well depend on regular and continuous therapy. An irregular treatment schedule resulting in sub-optimal dosage may significantly reduce the drug's therapeutic efficacy, though as no biomarkers are available to estimate therapeutic efficacy, the effect of suboptimal dosage over time on the progression of the disease is difficult to estimate.

In short, repeatedly missed ERT may jeopardize therapeutic efficacy and put patients at risk. The subsequent lack of positive effects from irregular ERT may also potentiate patients' feeling of the treatment being cumbersome yet inefficient. In addition, within the clinical setting, patients' reduced cooperation with the therapeutic schedule may become a source of potential tension between health care providers and patients. Medical professionals might perceive non-adherence to ERT as a “waste” of considerable health care resources for “a non-cooperating” patient, as well as displaying a disregard for their efforts to provide adequate care. Patients, on the other hand, may not always understand the medical importance of the treatment and the implications of missed ERT, and may thus perceive any discussion about their “cooperation” as unjustified.

In spite of these obstacles to care, there has been no research into the illness perception and treatment experiences of patients with MPS VI and the ways in which their therapeutic experiences affect the course and acceptance of therapy. This issue may be even more important in patients with a migration background, as they may experience a double exclusion in wider society as well as within the health system: i) due to having a severe illness, and ii) because of their migration background.

This study was conducted through cooperation between the Institute of Social and Cultural Anthropology at the Freie Universität Berlin and the Centre of Excellence for Rare Metabolic Diseases at the Charité-Universitätsmedizin Berlin. We focused on the following issues:

- What are patients' understandings of and knowledge about MPS VI and how do they experience ERT?
- How are these aspects influenced by the relationship between health care providers and patients?
- To what extent do cultural factors (such as culturally shaped understandings of the disease or religious beliefs) influence the therapy and illness perception?
- How do social aspects such as education, social background, and family relationships determine the health behavior of MPS VI patients?

Over the course of five months, an ethnographic study was conducted investigating patients' knowledge of MPS VI and their perceptions of the clinical treatment at two locations in Germany: i) the Center of Excellence for Rare Metabolic Diseases, Charité, Berlin, and ii) the Department of Pediatric and Adolescent Medicine, Villa Metabolica, University Medical Center, Mainz. The study also examined the effects of structural differences, i.e. adult patient versus pediatric care, on the relationships between patients and health care providers in these two settings. The qualitative methodology allowed for determining the social, individual-biographical, and cultural aspects of illness and therapy perception.

The first part of the article explores the theoretical framework and explains some of the anthropological concepts used in the study. Subsequently, the methods and the limitations of the research are presented. The results are subdivided into patients' socio-demographic backgrounds, their knowledge of MPS VI, their perception of ERT, the relationships between patients and health personnel, and the influence of cultural and social determinants on illness and therapy perception.

The investigation was performed according to the ethical rules of the institution referring to the Declaration of Helsinki and Good Clinical Practice, as well as to the respective EU regulations. All patients signed an informed content. In addition the aims and methods of the investigation were individually discussed with the patients.

### Theoretical framework

One fifth of the total German population has a migration background, but insufficient information is available about their health conditions and experiences [15]. Few medical or qualitative studies have explored the relationship between the health status and health behaviors of migrants in Germany by including epidemiological as well as social, cultural, and economic factors in the analysis [16]. This may be due in part to some basic difficulties encountered by such investigations. First, the definition of the category of “migrant” often differs among studies. Apart from the term “migrant,” other designations, such as “resettler,” “foreigner,” or “native speaker,” are used [16]. Thus, there is no consensus on the actual groups to which health-related studies refer [15].

Second, many people described as migrants have had no personal migration experience, were born in Germany, and have German citizenship. This does not mean, however, that their everyday lives and health experiences are not shaped by the fact that their parents or grandparents settled in Germany as migrants. Until the year 2004, only those without German citizenship were officially recorded as “migrants” in Germany. It was not until 2005 that the Federal Department of Statistics introduced the category “people with a migration background,” which includes naturalized Germans, their children, “Spätaussiedler” (ethnic German repatriates), and children from bi-national marriages [17]. The Robert Koch Institut adopted this definition and comprehends people with a migration background as: i) individuals who have migrated themselves, ii) someone with one parent who was not born in Germany, or iii) someone for whom both parents immigrated to Germany and/or do not have German citizenship [18].

Finally, statistical discrepancies may appear that shed critical light on previously conducted epidemiological studies on health and migration. For example, in some cases, even when older migrants return to their home countries, they still remain officially registered in Germany, rendering unfeasible the gathering of reliable mortality statistics [19].

With regard to qualitative research, the risk of overemphasizing the “cultural background” of migrants in studies on health behaviors as well as clinical settings has been highlighted by medical anthropologists. If a migrant's health behavior is interpreted as being culturally determined, communication (linguistic as well as cultural) and translation problems are likely to be foregrounded and notions of segregation and cultural difference may be reinforced [20] [21]. This can lead to stereotyping, while other important determinants such as socio-structural and individual-biographical factors are not taken into consideration. Furthermore, while cultural competency is no doubt required, health care providers should be aware that “there is something more basic and more crucial than cultural competency in understanding the life of the patient, and this is the moral meaning of suffering–what is at stake for the patient; what the patient, at a deep level, stands to gain or lose” [22].

Building on the insights of existing anthropological and migration-related investigations, this study assumed that health and illness behaviors are influenced by the subjective perceptions and experiences of patients as well as their social, cultural, and individual-biographical backgrounds. In medical anthropology, the patient's subjective experience of ill health or adverse health events is described by the term *illness*. It differs from the concept of *disease*, which is based primarily on the physical-pathological dimensions of an ailment [23]. Conscious of this distinction, this investigation specifically asked whether the socio-cultural factors of patients with a migration background influenced their subjective experiences in relation to their illness and health situation, as well as their coping practices with regard to the biomedically defined disorder.

Biomedical practice is, furthermore, a cultural system “of symbolic meanings, anchored in particular arrangements of social institutions and patterns of interpersonal interactions” [23], involving negotiation processes between patients and health professionals that take place in the context of social power structures [21]. This has an impact on the treatment situation and on physician-patient communication. Therefore, this study analyzed the interaction between patients and medical personnel and the resulting treatment practices, in connection with the structure of the medical institution. This included, for instance, comparison of the availability of time and health staff in a setting of adult patient care (Berlin) versus pediatric patient care (Mainz).

### Methods

An ethnographic study was conducted over a period of five months (May until September 2011), applying the following methods:

1. Throughout the study, the core method of anthropology, participatory observation, was used. This means that the clinical situation, especially the procedure surrounding ERT, consultations, and treatment encounters, were observed and analyzed, both in the Center of Excellence for Rare Metabolic Diseases (hereafter CRMD) in Berlin and the Center for Lysosomal Storage Disorders (hereafter CLSD) at the Department of Pediatric and Adolescent Medicine, Villa Metabolica in Mainz. The information from the observations was logged in a field diary. The research also applied direct observations in the homes and everyday lives of four patients in Berlin in order to examine their living situation as well as their family and social networks. This allowed for a deeper insight into the social and (potentially) cultural or religious determinants of the patients' health behaviors and therapeutic experiences.
2. Semi-structured interviews were conducted with:
  - Eight representatives of the health care providers at the CRMD, Berlin (average length 30 minutes), in order to examine their perspectives about patient cooperation (see Appendix S1).
  - Six Turkish patients (four in Berlin, two in Mainz), to explore their clinical treatment experiences and the ways in which they cope with the illness in their everyday lives.
  - Four parents (three mothers and one father) of Turkish patients, as these four patients were too young to be interviewed.
    For the interviews, a narrative approach (focusing on people's story-telling about their life experiences) was applied for the following reasons:
  - Individual illness and therapy perceptions are dependent on a range of factors (treatment situation, educational background, family environment, etc.), and the interplay between them cannot be explored using standardized methods such as questionnaires.
  - Due to the low prevalence of MPS VI in Germany, a quantitative research would not have produced reliable results.
    Based on the clinical observations and preliminary conversations with patients and medical staff, two interview guidelines – one for health personnel (see Appendix S1) and one for patients (see Appendix S2) – were developed and tested. The guidelines for patients included questions regarding biographical and socio-cultural aspects as well as the migration history of the patients' respective families. The data gathered during the patient interviews gave insight into how individual patients interpreted their illness and perceived the therapy, and allowed for comparison between patients. With one exception, the interviews with patients or their parents (average duration 45 minutes) were held during their weekly ERT infusion. As all patients had very good German language skills, the interview language was German. Due to the age of the patients in Mainz (7 to 23 years), in four cases only their parents were interviewed. As especially the parents in Berlin had poor German language skills, informal conversations during participatory observation in the patients' homes switched between German and Turkish. All of the interviews were recorded with the verbal consent of the interviewed patients, parents, and health care providers.
3. Throughout the period of data collection, medical records and the relevant medical and anthropological literature were reviewed.
4. For analysis purposes, the interviews were transcribed. Afterwards, the interviews as well as the medical and personal records from the field diary were coded by identifying the relevant aspects of the research, cross-related, and finally analyzed.
5. The patients were recruited from the two largest centres dealing with inborn errors of metabolism in adults in Germany, Berlin and Mainz. Inclusion criteria were a genetically confirmed diagnosis of MPS VI, migration background and willingness to participate in the interview. No exclusion criteria were defined. For retrospective data collection ten patients gave their informed content by signing the treatment contract with the University Hospital. The contract states that the patient agrees to have his/her anonymized clinical data analysed for research purpose. No further ethic application is needed in this case. For the interviews the patients or their respective parents were informed about the goals of the investigation and asked to consent to the interview. This was documented in the patients' file.

The Ethical Guidelines of the Charité require no ethic application in case of an interview. The investigation was performed according to the Declaration of Helsinki (1964 and amendments up to 2008), the Guidelines of Good Clinical Practice (GCP 2004) and the Ethical Guidelines of the Charité. All the patients interviewed in this study gave their informed consent to the investigation. The investigation was performed according to the Declaration of Helsinki (1964 and amendments up to 2008), the Guidelines of Good Clinical Practice (GCP 2004) and the Ethical Guidelines of the Charité.

### Limitations of the study

The observations in Mainz and Berlin differed in terms of intensity and duration. The observations in Mainz were conducted over a period of one week, while those in Berlin took place over more than one month, and unlike in Berlin, the social environments of the patients in Mainz were not observed. Furthermore, interviews with health care providers were only conducted at the CRMD in Berlin. Thus, the clinical situation in Berlin was analyzed more extensively.

The two centers also differed in terms of the type of patients cared for. While the CRMD in Berlin treats only adult patients with metabolic diseases, the CLSD in Mainz is a pediatric unit where the patients' parents are very much involved by the care providers. The patients involved in the study thus differed in terms of age and the level of personal responsibility that they exercise over their therapeutic situation.

Finally, the researcher's (L. Leissner) social background, ethnic identity, as well as personal concern regarding the emotionality of the medical issue, were a challenge for the data collection. The following issues were of relevance:

- It could be assumed that a researcher with the same cultural background (in this case, of Turkish origin) would have experienced a greater attitude of openness compared to a white German researcher in terms of the information given by the patient participants. However, this assumption was thwarted by a previous experience at the CRMD in Berlin, where a specifically recruited psychiatrist with a Turkish background was unable to establish in-depth communication with the MPS VI patients. This led the authors of this article to the assumption that the overall hierarchical nature of the therapeutic setup, in which the anthropological researcher entered, had an equally strong impact on the research encounter and the collected data as the researcher's own cultural background.
- The encounter with people with a severe debilitating disease personally challenged the researcher, who is not trained as a medical professional, and so, to a certain extent, was concerned by the fear and despair expressed by the patients and their parents. Sometimes sympathy and solicitousness was verbalized by the researcher during the interviews, and thus the possibility of this having had an influence on the answers of the interviewees and on the data collection in general cannot entirely be ruled out.

## Results

### Medical, social, religious, and demographic backgrounds of the patients

Of the ten patients involved in the study, three were male and seven were female. The age-span of the participants was between 7 and 31 years (Table 2) [24] [12].

**Table 2:** Patients' social, religious, and demographic backgrounds.

Person	Gender	Age	Location	Religion	CP	Educational level	Work
1	male	18	Berlin	Muslim (Alevi)	yes	Primary school	unemployed
2	female	26	Berlin	Muslim (Alevi)	yes	Primary school for disabled people	working
3	male	27	Berlin	Muslim (Alevi)	yes	GCSE	unemployed
4	female	31	Berlin	Muslim (Alevi)	yes	GCSE	unemployed
5	female	7	Mainz	Muslim	no	Primary school for disabled people	n.a.
6	female	9	Mainz	Muslim	yes	Primary school for disabled people	n.a.
7	female	13	Mainz	Yazidi	no	GCSE	n.a.
8	female	16	Mainz	Muslim	no	GCSE	n.a.
9	female	18	Mainz	Muslim	yes	GCSE for disabled people	n.a.
10	male	23	Mainz	Muslim (Alevi)	yes	GCSE for disabled people	unemployed

CP - consanguineous parents.

GCSE - General certificate for the secondary educationn.a. - not applicable.

The patients were all born in Germany and had similar social and economic backgrounds. In the course of the labor migration of the 1960s, the patients' grandparents had come from urban and rural areas in Turkey to workforce-demanding regions of West Germany. The patients' parents had subsequently found work in the manufacturing industry or in the service sector. In one case the father went to university in Germany. At the time of the investigation, in four cases both of the patients' parents were unemployed. Five of the patients were aiming for a General Certificate of Secondary Education (GCSE) or in the case of three adults already had one. One was just about to quit school without a GCSE degree and another had stopped school after finishing the primary school. Five patients went to schools for physically disabled people. Two of the six adult patients had finished an apprenticeship but were unemployed at the time of the research. Only one woman worked (in a workshop for disabled people). The interviews showed that the patients experienced little social mobility in comparison to the previous generation.

Nine patients were Muslim, of which five were Alevi, and one patient was Yazidi. Two patients lived in a strongly religious household, while the others practiced religion only on an irregular basis. All of the four Berlin patients and one patient in Mainz were children of consanguineous marriages, and all were related to each other in second or fourth degree – these were the five Alevi patients.

Only one female patient was living in her own household with her husband and two children. The other patients had no children and were living together with their parents and siblings.

### Patients' knowledge about MPS VI

This section explores the knowledge of the patients involved in the study about the etiology and medical implications of MPS VI.

The patients were diagnosed with MPS VI at a median age of 6 (0–26) years. ERT was initiated at a median age of 13 (4–26) years, with a mean interval of 8 (0–12) years after diagnosis. Eight of the ten patients are considered to have a severe form of the disease. ERT had normalized urinary glycosamin concentration at last follow-up in seven of the ten patients. Despite this, all patients demonstrated slowly progressive deterioration.

All but one patient had been diagnosed during childhood and thus the medical nature of MPS VI had been explained to their parents. Thereafter, the four adult patients in Berlin claimed that no further (formal) medical education had been given, neither by health care providers nor family members. This was confirmed by two of the interviewed medical doctors in Berlin:

*No, I have not done it [explaining MPS VI to the patients]. We always discussed the individual diagnostic findings and things like performance and pain but about the disease from the outset, no we have not talked with them about it.* (Medical doctor, Berlin)

*Yes of course, in concrete terms you always explained to them what they are suffering from, which complications exist, why you are doing the treatment and so on. And when they are always nodding and saying ‘yes, yes,’ you think that they have understood and you assume that they know, because you have not done the first diagnosis. If they [the patients] come from a diagnosis and a treatment, you don't start to explain everything from the beginning. Probably it's wrong but, I don't know, if they were informed when they came here for the first time, but maybe that is not sufficient. You assume it.* (Medical doctor, Berlin)

Consequently, with one exception, the patients and their parents in Mainz and Berlin had only marginal knowledge about the biological causes and repercussions of the disease:

*I can't describe to you what MPS is.* (Patient, 27, Berlin)

Four patients and two parents did not know that the disease was inherited. Furthermore, the patients and their parents had only a vague understanding of what an inherited disease actually meant:

*Yes, it's coming from the family, but I don't know why.* (Patient, 27, Berlin)

Most patients could only describe a few symptoms:

*Honestly, right now, I don't know. I can't describe it myself, I'm only 18 and at 16 I just started to get informed. (…) I have problems with the blood, as you know, and I have lung problems. Heart problems I do have. And back pain a lot, I get very confused and, I don't know, with which problems to start.* (Patient, 18, Mainz)

Five adult patients highlighted that they “do not want to understand their disease,” because “it wouldn't change anything.” This phenomenon was observed explicitly among the patients in Mainz and Berlin who belonged to the same family:

*I've never talked with them [the health staff] about it. He [the doctor] tried a few times to explain, but I've never wanted to understand what I'm suffering from.* (Patient, 23, Mainz)

*You never wanted to understand what you are suffering from?* (Interviewer)

*No, I didn't, no. For what reason? I have it and you can't change it anyway, it won't get any better.* (Patient, 23, Mainz)

Taken together, the patients' knowledge of this complex disease and its consequences is poor.

### The perception of ERT and patient cooperation

The not knowing and not wanting to know about MPS VI had a particular impact on the perceptions of patients concerning the treatment and clinical situation. This section describes the advantages and disadvantages of the therapy from the point of view of patients and their parents.

In Berlin and Mainz, the MPS VI patients complained about the duration and frequency of ERT. It has to be kept in mind that in addition to ERT, the care for patients with MPS VI includes other regular appointments, such as physio- or speech therapy on a weekly basis, as well as extensive routine follow-up investigations once or twice a year such as MRT, ECG, echocardiography, neurology, etc.:

*To come here every Tuesday bothers me. It annoys me (…). If I'm not in the mood, I call and say that I won't come.* (Patient, 23, Berlin)

*At 07:40 a.m., I was here and I had to wait two hours for nothing. Sometimes it bothers me.* (Patient, 26, Berlin)

Often, the child patients have to miss an entire day of school due to the up to 5 hour long treatment sessions. The therapy poses a major challenge and mental stress for their parents, too, because they are forced to organize their everyday lives around the numerous treatments, appointment, and follow-ups:

*Let me put it this way, the therapy itself doesn't bother me, but coming here every week bothers me. After some years, it causes nervous breakdowns, I already had some.* (Mother of a patient, 7, Mainz)

*She loves going to school, so it's an extreme burden, that she has to go to the hospital every Thursday and so she can't attend the lessons.* (Father of a patient, 13, Mainz)

From the patients' point of view, the initiation of therapy often increased hopes and expectations for physical improvement. This, together with the lack of insight into the character of the disease, resulted in a significant disappointment when they perceived only a subsequent stagnation in their health situation. This was expressed by several patients, for example:

*Well, I was fit, so I was thinking, it [the therapy] will be more effective, and then after some time, I found out that it won't get better, and it won't get worse, it will just stay the way it is. Then I started not to go.* (Patient, 23, Mainz)

In general, there were many diverse perceptions among the patients regarding the extent of the change in their health condition brought about by ERT. In particular, this varied in terms of the progress of the illness and the age of the patient. The younger patients in Mainz maintained an overall positive attitude towards the perceived medical effects of the therapy:

*In the beginning I recognized that I'm not sick so often anymore and I grew some centimeters.* (Patient, 18, Mainz)

In contrast, all patients in Berlin claimed to be entirely unaware of any therapeutic benefit:

*My mother said I got better from the therapy. Personally I haven't noticed it.* (Patient, 26, Berlin)

What was common to all the patients in Berlin and Mainz was that they were not able to explain what kind of medication the infusion contains:

*I really don't know what's in there. (…) What kind of medication and so on, what it does. They said, it's good for bones, ears, eyes, but I don't know exactly. (…) They explained it to me, but I've never kept it in mind. I didn't care. (…) I think I listened a little bit, and a little bit not.* (Patient, 27, Berlin)

The Berlin patients did not attend ERT regularly – not only because of the long duration of the infusion, but also because of its perceived “ineffectiveness.” The overall rate of missed therapy sessions among the four MPS VI patients in Berlin in the period from January to September 2011 was 38%. The health care providers in Berlin assumed that the therapeutic impact would decrease with low infusion frequency, and thus expressed concern that this would merely incur high health care costs without benefits for the patient.

Taken together, the participating patients and their parents' insight into the medical-technical aspects of MPS VI and ERT is poor. For several patients, especially those belonging to the same family in Berlin, the treatment was perceived as ineffective and cumbersome. Overall, awareness of therapeutic efficacy and adherence to the treatment sessions were negatively correlated.

### Relations between patients and health care personnel

This section describes how the relationships – in terms of interaction and communication – between the health care providers and patients affected the experience and perceived outcome of MPS VI treatment.

The CRMD is part of the Interdisciplinary Centre for Metabolism at the Charité, Berlin. It is a University Outpatient Clinic for adult patients who are cared for by two specifically assigned physicians. For ERT, the MPS VI patients are referred to the department's day care clinic. There, physicians and nurses are responsible for a broad range of patients, with little additional time at their disposal for informal conversations with individual patients. The MPS VI patients' supposed main contact is the physician at the outpatient clinic, who is responsible for their medical care. Based on an informal conversation with medical doctors at the CRMD, the clinical interactions with adult patients aim for: i) “empowerment” of the patient, allowing for a responsible interaction with an informed patient, as well as ii) a “non-paternalistic relationship” between the health care providers and patients. All MPS VI patients at the CRMD were transferred to the adult outpatient clinic via a standardized transition from the Metabolic Department of the Otto-Heubner Centre for Pediatrics, Charité, Berlin.

The CLSD is a Metabolic Unit of the Pediatric Clinic at the University of Mainz. The physicians and nurses are highly specialized in the care of metabolic diseases. According to the needs of children the patient-staff ratio is more favorable than in Berlin. While the institution is set up specifically to care for children, due to the regional lack of metabolic care for adults, patients at the Pediatric Clinic are cared for well into their adult life. The interviews with the patients and their parents in Mainz underlined the special trust relationship that is built up with the medical staff, as many have been in long-term treatment since childhood.

In the CLSD in Mainz, the general organizational structure of the facility enables extensive doctor-patient consultation, which goes beyond purely medical care. Both a nurse and a physician are exclusively available for the MPS VI patients. Many of the patients in Mainz have been there since their first diagnosis, and in case of problems and questions, they contact the clinic first. For patients and their relatives, it was important to have the feeling of being able to find help at any time:

*If I have any questions, concerns, problems, I can call there at any time and everybody knows, who is on the phone.* (Mother of a patient, 7, Mainz)

In Berlin, the relationship between medical staff and patients was characterized by a greater personal and emotional distance than in Mainz. The health professionals perceived patients' attitudes during the consultations as passive and claimed that they just “spoke on demand.” Furthermore, all patients in Berlin were perceived to be reluctant to talk about personal matters:

*I have the feeling the conversations do not go well, the answers are very short and they don't talk much, how they feel etc.* (Medical staff member, Berlin)

During the observations of the treatment situation in Berlin, the researcher also witnessed the patients' “silence.” The reason given by these patients for this silence was their fear of saying “something wrong.”

In Mainz, on the other hand, the structural conditions at the CLSD – i.e. the long-term care from childhood into adolescence and the better ratio of medical personnel to patients – resulted in individualized care and continuous support for both patients and their parents.

### Social and familial influences on illness and therapy perception

Apart from the structural conditions of the clinics, the experience of the illness and the therapy were also affected by the wider social and familial circumstances of the patients. During the home observation visits in Berlin, it was noticed that the subject of the disease was not brought up. The parents did not talk about it. In one case, the mother did not even want her eighteen year old son to know about his disease. The siblings of all patients in Berlin were also not informed about what disease their brother or sister was suffering from. Even the patients themselves did not share their experiences and feelings regarding MPS VI with their family members.

*I'm used to it, because at home we don't talk, outside I don't talk, also at work. I also don't want to speak about it.* (Patient, 26, Berlin)

*In our family problems don't exist, we just suppress them.* (Patient, 23, Mainz)

One patient expressed the reasons for this “silence”:

*Because I want to forget, I want to live, how I am now.* (Patient, 27, Berlin)

In Mainz, where in most cases the parents of the MPS VI patients were interviewed, a more open handling of the disease was noticed:

*We are talking openly about it, because I said, I don't lie to her. Why should I hide it from her? She has to grow up with it and finally she has to live with it.* (Mother of a patient, 7, Mainz)

Taken together, it was observed that the way in which patients' families handle the disease significantly affects the patients' perception of MPS VI and hence their acceptance of the therapy.

## Discussion

This research contributes to current studies on migration and therapeutic experience among patients with a rare disease such as MPS VI [9] [25]. It shows that in addition to patients' individual knowledge and perception of the disease and its treatment, it is important to analyze the social and family backgrounds of patients, as well as the structural situation of the medical facilities in which they are treated.

Until the introduction of ERT with recombinant human ASB arylsulfatase (rhASB, galsulfase, Naglazyme ®) in 2006, the only available therapy for MPS VI patients was symptomatic treatment. Results from a double-blind, placebo-controlled, multi-centre study with galsulfase [26], and other smaller studies, have demonstrated improvements in the 12 minute walking and stair climbing test [27] [28]. In addition, reduction in liver size, objective improvement in general well being, and reduction on the pain score have been reported [28]. However, as biomarkers reflecting therapeutic efficacy are still lacking and final endpoints take years to develop, the definite benefit of ERT is still a matter of debate [29]. Complete or even partial remission of all changes induced by lysosomal storage and the subsequent pathological processes are not expected. MPS VI is a progressive disease and at best ERT may predominantly provide stabilization, preventing further aggravation of the condition.

Aside from the medical evidence of the results of ERT, therapeutic efficacy is greatly influenced by patients' expectations and acceptance of the therapeutic regimen. Yet so far there has been no research on the illness perception and treatment experiences of patients with MPS VI, and on the ways in which their therapeutic experiences affect the course and acceptance of therapy. This study used an interdisciplinary approach by integrating medical and anthropological aspects and demonstrated that patients' behavior vis-a-vis the therapy and how they cope with their illness can be explained by an interplay of various factors.

### Patients' knowledge about MPS VI

The patients' knowledge about the disease was generally poor, as was their understanding of the ERT. MPS VI is a complex disease and difficult to understand for persons with a non-medical professional background, even with repeated information given to patients about the disease, therapy, and the need for follow-up. Two principal reasons for this knowledge deficit in the patients could be demonstrated: firstly the lack of encompassing information about MPS VI and ERT given by health personnel and secondly because of the patients “not wanting to know” about their illness. Furthermore, in the clinical setting in Berlin, even when medical aspects were not fully understood, the patients did not dare to ask. They seemed intimidated by the difference in status between themselves and the medical staff and the acknowledgement that they probably did not “correctly” understand the medical information. This may have resulted in a self-perception of inferiority in relation to the health care providers. The analysis below shows that this “silence” concerning and the “not-knowing” about MPS VI is closely related not only to the therapeutic conditions in the clinical settings, but also to social aspects, for example how the disease is managed within patients' families.

### The perception of ERT and patient cooperation

Medical aspects play an important role in terms of cooperation and illness perception. The clinical picture, such as like the severity of symptoms, determines significantly a patient's self-perception in relation to their illness of the patients. When the patients in this study suffered from few symptoms and the disease was morphologically less visible, they often perceived themselves as healthy and of having only minor constraints in their everyday lives. Therefore, if the conditions of the therapy – such as the frequency, duration, and notable efficacy of ERT – are perceived as a burden, this can reduce therapy acceptance. On the other hand, even if patients *do* experience many severe and visible symptoms, and perceives themselves as ill, the lack of subjectively measureable improvements brought about by the burdensome ERT schedule may also have a negative impact on therapy acceptance.

### Relations between patients and health care personnel

The structure of the health care facility was found to have a significant impact on the patient-physician relationship, and consequently on the perception of, as well as the cooperation with, therapy. Time and staff availability in the hospitals are crucial in order to provide successful treatment and build up a relationship of trust with patients. The example of the CLSD in Mainz demonstrates that due to the individually focused and extensive care of patients, therapy acceptance is high. On the other hand, the lack of both time and health care personnel in the CRMD in Berlin had a negative impact on patient cooperation. It has to be kept in mind, however, that in the pediatric facility in Mainz it is the parents who are responsible for keeping up with the therapeutic schedules, thus treatment adherence is maintained by someone who is only indirectly affected by the disease or the therapy itself, but who is also ultimately responsible for the young patient's health. In contrast, in Berlin the responsibility for treatment adherence lies with the individual adult patient. This may well partly explain the difference in adherence to the ERT treatment schedule in favor of Mainz.

### Social and familial influences on illness and therapy perception

Not only the structure of the health care setting but also the family background is significant for the ways in which patients manage their disease. These two aspects may also interfere with each other.

Marschalck and Wiedl [30] argue that patients' social network, in this case the family, has an important influence on the way in which patients deal with the disease, though this is not always in a positive way. This finding is consistent with Mark Nichter's research on the “social relations of therapy management,” in which he states that: “The study of therapy management entails not only what people do (and can do) and reasons for actions taken, but what they are unable to do and what lies under apparent passivity, acceptance, or fatalism” [31]. The “silence” about MPS VI within the family in Berlin resulted in a suppression of discussion and a passive handling of the disease. It explains why the patients did not want to understand their illness [32]. In this case, not wanting to know can be a sign of a particular illness perception and illness behavior, and further it can be assumed that this aspect will reduce the willingness of patients to accept treatment. In particular, patients' insistence on “not wanting to know” may be perceived in the medical encounter as a “wall of silence,” and the health care providers may therefore experience difficulties in building up a relationship of trust and communication with these patients.

Unfortunately, due to the small number of patients in the study, we were not able to arrive at a definitive conclusion concerning the effect of the family and health care structures on the perceptions of and ways of dealing with MPS VI and ERT – and the way these individually reported perceptions and ways of coping do (not) reflect a larger cultural pattern. In particular, since all four of the patients in Berlin (and one in Mainz) were from the same family, some of the challenges faced in Berlin may well be related to the specific socio-familial dynamics among this group of patients where the practice of intermarriage, especially between cousins, existed. Thus, the physicians in Berlin reported that there are currently about eleven cases of MPS VI in this one family. According to family members, the other five relatives with MPS VI live in Turkey. Probably as a defense mechanism to cope with shame and guilt about the disease, the family members did not talk about it. This self-protection has likely developed out of fear of stigmatization of the family. The fact that cases of MPS VI had accumulated within the family over generations, combined with mutual loyalty and the shared fear of outside stigma, have led to silence about the illness as a dominant way of coping among family members. This is supported by the fact that even though all but one of these five related patients were severely affected by the disease from a medical perspective, it was important for them to be perceived as healthy by others and to have a “normal” life.

Furthermore however, the dynamics of “silent coping” *may* have been reinforced by the fact that the family members in Berlin and the patient in Mainz have an Alevi background. In the Muslim world, Alevis are often exposed to the accusation of “incest” and their affiliation to the Islamic community is denied [33]. This particular situation may possibly increase these patients' experience of double marginalization: due to their status as migrants and within the larger Muslim community on the one hand [34] [35] [36], and as MPS VI patients on the other.

The way in which problems are dealt with, or rather not dealt with, within this family may therefore be a coping strategy induced by their marginalization, as well as by a feeling of shame and guilt that has been expressed by some family members [37]. This family strategy of avoiding the problem of MPS VI may well shape the ways in which the patients themselves handle their disease. Thus the familial (and potentially also the religious) background of these patients has to be taken into account and it is evident that additional psycho-social support may be needed to allow for effective medical therapy.

### Perceptions of health care providers

It is of interest to note that the health care providers in Berlin were aware of the particular difficulties in dealing with this group of patients. However, they perceived the patients as “foreign,” and thought that an anthropological investigation would be necessary in order to understand them. According to their understanding, an in-depth investigation into the “intercultural problems” of these patients would possibly help both the patients and the health care providers improve their mutual cooperation. However, if a migrant's health behavior is interpreted solely as being culturally determined, this is likely to lead to stereotyping, while other important determinants such as socio-structural and individual-biographical factors are not taken into consideration [20]. In the case of the Berlin family, social and family structures – *possibly* reinforced by their cultural background as Turkish Alevis – were more important in shaping the patients' health care behavior than the fact of their migration background per se.

**Figure 1:**
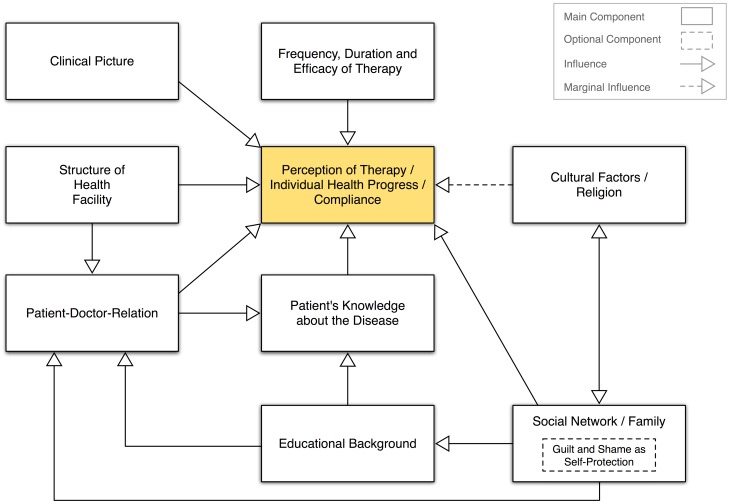
Determinates influencing individual health progress (source: authors).

In summary, medical and social components, such as family background and the structure of health facilities, had an important yet varying impact on patients' therapy perception and individual health attitude. Our data show that cultural factors (such as religious background) may have reinforced specific ways of coping with MPS VI illness, but were not necessarily central to the ways in which patients handled the disease, and could be neglected in the analysis (see Figure 1). Finally, the influence of educational background on patients' knowledge about MPS VI may well have shaped their subjective illness perceptions and the patient-physician relationship. This latter aspect needs to be explored in further comparative research with larger samples of interviewees.

To conclude, the following four aspects could lead to an improvement of the clinical situation for people with a chronic disease such as MPS VI, and thus increase the willingness of patients to receive therapy and improve the relationships between patients and health professionals:

- The lack of knowledge among MPS VI patients about the disease has important consequences for the perception of the therapy and the clinical situation. Patients and their families need to be informed about MPS VI during the therapy on a regular basis, because patients' perceptions about the disease shift over time. There should be specific courses for patients that include family members, held by Turkish speaking health personnel, where appropriate, because – in contrast to the patients in this study – especially the parents in Berlin had usually insufficient German language skills. The relatively low educational level of the patients and their parents could be one reason why they had such minimal knowledge about MPS VI, but due to the small sample size, further research is needed to verify this hypothesis. Nonetheless, with regards to the relating of clinical and disease-related information to patients, the educational background of patients should be taken into consideration and an appropriate didactic concept should be applied.
- Despite the fact that German hospitals face financial restrictions, the recruitment of additional nursing staff to care for patients with a chronic condition should be considered, in order to ensure the best care possible. In this regard, health providers should respect the patients' cultural and social background and their personal perception of the disease and the therapy. Yet structural and social aspects (clinical setting, family and educational background) may be even more crucial here than “cultural barriers.”
- The number of doctor-patient discussions should be increased to build up a relationship of trust. The setup, in which a layperson meets the expert in a consultation, may lead to the patient feeling intimidated during the interaction. While this is potentially true for every patient-physician interaction, it may have a greater impact in interactions with patients who feel themselves already marginalized due to their disease, migrant status, and lack of formal education. Health care providers should be aware of their status as “respected persons” and should try to adapt to the educational background of patients during the communication of medical details. Health care providers should also acknowledge that MPS VI patients suffer from mental distress. A foundation of trust for successful therapy is therefore essential.
- To understand patients' clinical experiences and illness perceptions, this study concludes that further interdisciplinary research, including anthropological and medical aspects, should be conducted to improve the treatment of patients with a migration background suffering from rare metabolic diseases. In particular, future research should pay more attention to the influence of a patients' social network, especially the family, on illness and treatment perception, as well as its impact on the therapeutic outcome.

## Supporting Information

Appendix S1 Interview Guideline – Medical Staff (Berlin).
(DOCX)

Appendix S2 Interview Guidelines – MPS VI Patients with a Turkish Background.
(DOCX)
