# Supplementary material for: Illness Perception and Clinical Treatment Experiences in Patients with M. Maroteaux-Lamy (Mucopolysaccharidosis Type VI) and a Turkish Migration Background in Germany
Source: PLoS One. 2013 Jun 24;8(6):e66804. doi: 10.1371/journal.pone.0066804 (PMC3691296; doi:10.1371/journal.pone.0066804)
Supplement: Appendix S1 — Interview Guideline – Medical Staff (Berlin). (DOCX) [file pone.0066804.s001.docx]

Appendix S1:

Interview Guideline – Medical Staff (Berlin)

1. Since when have you been working at the Metabolic Center? Have you been working in other departments before?

2. What is your work?

3. Have there been any major changes within the Metabolic Center recently? If so, what has changed? What kind of effects did these changes have a) on the work at the department b) for the patients?

4. According to your knowledge, how does the enzyme replacement therapy (ERT) affect the condition of the patients? How do you communicate the effects of the therapy to the patients? Did you notice any challenges in communication (give examples)?

5. Can you describe how MPS patients with a Turkish background respond to the therapy? How does the patient’s behavior itself affect the therapy progress / the therapy application?

6. Did the attitude of the patients towards the therapy change in the last years? If so, how?

7. To what extent does the attitude and handling of the disease / of the therapy among patients with a Turkish background differ from other patients receiving enzyme replacement therapy? Are there any special challenges for them?

8. In your opinion, what are the reasons for these kinds of differences in handling the therapy / the disease?

9. To what extent does the fact that all patients with a Turkish background come from the same family affect their handling of the disease / the therapy?

10. In your opinion, do cultural practices influence the way people handle the disease / the therapy? If so, how?

11. What do you know about the family of the patients? What do you know about the internal family relations?

12. How does communication with patients with a Turkish background take place? Did you notice anything special, e.g. in the conveying of treatment strategies? If so, what was it?

13. Do you see any need to improve the therapy situation? If so, which actions should be undertaken (have already been undertaken)?

14. How do you perceive the compliance of the patients with a Turkish background? Do you sometimes feel frustrated while working with the patients?

15. In the other departments you have been working in before, did you have any experiences with patients with a migratory background? If so, what kind of experiences? (Depending on question 1)

16. How would you describe the relationship among nurses and between the doctors and nurses?

17. How would you describe the work atmosphere at the Center?
