# Supplementary material for: Illness Perception and Clinical Treatment Experiences in Patients with M. Maroteaux-Lamy (Mucopolysaccharidosis Type VI) and a Turkish Migration Background in Germany
Source: PLoS One. 2013 Jun 24;8(6):e66804. doi: 10.1371/journal.pone.0066804 (PMC3691296; doi:10.1371/journal.pone.0066804)
Supplement: Appendix S2 — Interview Guidelines – MPS VI Patients with a Turkish Background. (DOCX) [file pone.0066804.s002.docx]

Appendix S2:

Interview Guidelines – MPS VI Patients with a Turkish Background

**1. General Facts: Bibliographical Facts / Social Factors**

1.1. From where did your parents originate?

1.2. When did they come to Germany?

1.3. What do you know about the life of your parents before you were born (profession/migratory experience)?

1.4. Where were you born and what can you tell me about your childhood?

1.5. Which schools did you go to? What did you do after you finished school?

1.6. Where are you living right now and with whom?

1.7. How would you describe your financial situation?

1.8. How many siblings do you have? What kind of profession do they have? Where do they live?

1.9. Where do your other relatives live?

1.10. Do you have contact with them?

1.11. How important is your family to you?

1.12. Are you and your family religious? Are you practicing? If so, what do you do?

1.13. What kind of relationship do you have to Turkey? Can you imagine to live there?

1.14. Do you like to live in Berlin (or other city) and why?

**2. Subjective perception of the disease in everyday life?**

2.1. How would you describe a typical day in your life?

2.2. Do you notice the illness in everyday life? If so, how?

2.3. How do people around you react to your illness?

2.4. Has your health condition changed over the years? If so, how?

2.5. Are you talking with others about your illness? If so, with whom? If not, why not?

2.6. Are you talking about the illness within your family? Are you talking about it with your relatives who are also directly affected by MPS?

**3. Knowledge about the disease**

3.1. Please describe, in your own words, what kind of illness you have.

Optional Question: Where does this illness come from? How did you come to realize that you have this illness? What kind of cure is there?

3.2. How do you explain that there are more of your family members who are affected by MPS?

3.3. When and how was the first time your illness was explained to you? By whom?

3.4. What did your parents exactly tell you about the illness?

**4. Experiences in the hospital / during the treatment**

4.1. What has been the progress of your illness so far? In which hospitals have you been?

4.2. Have you had any surgical procedures? If so, which ones?

4.3. In your opinion, what effect does the infusion have? How do you perceive that?

4.4. Is there anything that disturbs you regarding the therapy? If so, what? How could that be changed, in your opinion?

4.5. Are you attending therapy regularly?

4.6. If not, what are the reasons for you not attending therapy?

4.7. What are the effects if you do not attend the therapy?

4.8. How do you feel in hospital?

4.9. Do you have contact with other patients, too?

4.10. How would you describe your relationship with the doctors and nurses?

4.11. What do talks with the doctors look like? What do the doctors tell you? Do you always understand what they are telling you? If not, can you give examples?

**5. Personal health strategies**

5.1. What helps you if you are not feeling well?

5.2. What exactly do you do when you have pain?

5.3. Have you also tried other possibilities, besides the medication and infusion, to do something against the disease?

5.4. Have you tried alternative healing methods, too? If so, which ones? If not, do you know any of these methods?

5.5. Was there or is there any use of alternative healing methods in your family? If so, for what kind of illness/suffering?

5.6. Do you think that praying improves the disease or that religion can help in any other way?

**6. Final questions**

6.1. Are you satisfied with your life? Why?

6.2. What do you wish for the future of yourself and your family?
